# Validation study of the effectiveness of a stepwise care program for dementia family caregivers

**DOI:** 10.1002/alz.092289

**Published:** 2025-01-09

**Authors:** Jeewon Suh, Mi Kyoung Kang, Kyung‐hae Lee, Myoung Hwa Park, Im‐Seok Koh

**Affiliations:** ^1^ National medical center, Seoul Korea, Republic of (South); ^2^ Chungnam University, Daejeon Korea, Republic of (South)

## Abstract

**Objective:**

This study aims to validate the effectiveness of the education program for dementia family caregivers (so‐called Value Care program), and to assess the applicability of the program at local dementia centers in the community.

**Method:**

The Value Care program is an educational program designed for family caregivers of dementia, conducted once a week for 90 minutes over a total duration of 8 weeks. This program aims to enhance understanding of the characteristics and symptoms of dementia at various stages and to provide specific response strategies for caregivers.

In this case‐control study, family caregivers of dementia patients were recruited from 38 local dementia centers in Korea. Each participants were randomly assigned to either the experimental group or the control group in a 1:1 ratio. After 8 weeks, assessment was conducted on measure such as depression, care‐burden scales, caregiver self‐efficacy and satisfaction surveys.

**Results:**

A total of 226 family caregivers were recruited in the experimental group, and 210 participants in the control group. The experimental group showed significant reductions in depression (P<0.001) and care‐burden scores (P<0.001) and significant increases in caregiver self‐efficacy scores (P<0.001) after participating in the Value Care program. The overall satisfaction score averaged 4.47 over 5.

**Conclusion:**

The “Value Care Program” demonstrated effectiveness in reducing depression, care burden, and enhancing the efficacy of care for family caregivers of dementia patients. Participants expressed a high likelihood of rejoining the program and recommending it to others. Therefore, the Value Care Program could be a crucial initiative for alleviating caregiver burden, stress, and feelings of depression among dementia family caregivers.

